# Contingency response decision of network public opinion emergencies based on intuitionistic fuzzy entropy and preference information of decision makers

**DOI:** 10.1038/s41598-022-07183-7

**Published:** 2022-02-28

**Authors:** Sha Fu, Ye-zhi Xiao, Hang-jun Zhou

**Affiliations:** School of Information Technology and Management, Hunan University of Finance and Economics, No.139, Section 2, Fenglin Road, Yuelu District, Changsha, 410205 China

**Keywords:** Engineering, Mathematics and computing

## Abstract

A multi-attribute group decision-making (MAGDM) method based on intuitionistic fuzzy preference information is proposed for the multi-attribute intuitionistic fuzzy group decision-making problem where the decision-makers weight and attribute weight are completely unknown and the decision-maker has preference information for the scheme. Firstly, an intuitionistic fuzzy interval judgment matrix is established to describe the original data of the key decision indicators for multiple network public opinion emergencies that erupt simultaneously. Secondly, the attribute weights are determined based on the improved intuitionistic fuzzy entropy construction method, and the expert weights are determined by using objective decision information, taking into account the intuitionistic fuzzy entropy of decision matrix. MAGDM can not only synthesize experts' professional experience in different aspects, but also avoid experts' decision-making errors caused by insufficient domain knowledge. It is necessary to continuously adjust the expert weight based on decision-making information to make the comprehensive decision-making information more accurate. Thirdly, a scheme preference model and an attribute weight optimization model are established to determine the ranking method of intuitionistic fuzzy interval values. Then, a modified distance measure of intuitionistic fuzzy sets (IFSs) is introduced to make the evaluation result more accurate and reasonable when it comes to solving the deviation between the evaluation value and ideal solution of each scheme. Finally, the effectiveness and practicability of the proposed decision-making method are verified by an example of emergency crisis severity, It assists decision makers in selecting and implementing the optimal emergency response plan in a timely and efficient manner, which improves the emergency treatment efficiency of network public opinion crisis, helps emergency departments to better deal with the network public opinion crisis, improves the ability of public opinion guidance and control, and provides a new method and idea for multi-attribute intuitionistic fuzzy group decision-making problem.

## Introduction

With the increasing influence of network public opinion on the order of political life and social stability in recent years, some major network public opinion events have made people begin to realize the great role that internet plays in social supervision. Network public opinion, as a form of public opinion, is the highly influential and tendentious remarks and opinions of the public on some hot and focus issues in real life. The information of unexpected events is more likely to cause a variety of sentiments, attitudes and opinions of various social groups to spread in the network, and the network public opinion of such events is characterized by extremely strong complexity, variability and focusing. In recent years, the frequent unexpected crisis events, such as the explosion in Tianjin Binhai New Area, the stampede on the Shanghai Bund, the bus crash in Chongqing and the COVID-19 epidemic has posed a great threat to society and people's lives and property safety, and the sudden, urgent, serious, widespread and explosive nature of unexpected events have brought great challenges to the government and society. These large-scale emergencies have inflicted heavy losses to the economy, people's lives and property in the disaster-stricken areas. In the face of various frequently occurring natural disasters and emergencies such as earthquakes, tsunamis, malignant infectious diseases, and violent terrorist incidents, how to deal with them scientifically in a timely and effective manner has become a major topic that every country and regional government must face today.

Since the 1970s, the emergency response system for major emergencies has received widespread attention from the international community. Many industrialized countries and international organizations have successively formulated a series of emergency rescue regulations and policies, which clearly stipulate the responsibility and role of relevant government departments, enterprises, individuals and communities in the emergency response process, and corresponding emergency management agencies and government management departments are set up^1^. At present, in order to handle various emergencies, many countries and regions have established relatively mature coping mechanism against emergency management resource dispatching. For example, the United States, Russia, the United Kingdom, Japan, etc. have specialized agencies to cope with emergencies. China's research on emergency management started relatively late. The research on emergency management mainly focuses on the humanities and social sciences, with more qualitative and static researches, while quantitative and dynamic analyses are few. The complicated decision-making environment results in the emergency management department having to face a large amount of uncertain information, which makes it difficult to obtain sufficient decision-making information in a short time. If some negative information in network public opinion spreads freely on the Internet and cannot be effectively dealt with in emergency, the influence of individual emergencies will be infinitely magnified, which will have serious consequences for social harmony and stability and bring people life and property losses. As a result, the research on emergency decision-making of network public opinion emergencies has important significance and practical role. Media sentiments and group emergencies caused by social public opinion are no longer a single random event itself, but a systematic reconstruction of social public opinion communication environment, order and rules, which is a normal social existence that has an impact at any time^2^. Network public opinion has become an important form of social public opinion and has also had an impact on the decision-making of the government and the public sector. The research on the emergency decision-making of network public opinion emergencies has always been a hot topic in the academic field, and experts and scholars at home and abroad have actively explored this issue and achieved certain results. For example, Liu et al.^3^ explored the changes of netizens' opinions in network public opinion emergencies, constructed a topic map of network public opinion based on the knowledge map method, dynamically tracked public opinion, and comprehensively learned the development direction of network public opinion emergencies. Ma and Tu^4^ proposed a new knowledge map-based method for monitoring the hot spots of network public opinion in emergencies. Shao and Guan^5^ proposed an intelligent and participatory decision-making model, a process of "monitoring-early warning-decision-making" and a strategy of network public opinion data-driven decision-making. Wang and Li^6^ introduced evolutionary game theory to study the interaction between public opinion dissemination and emergency decision-making. On the other hand, the research on network public opinion emergencies presents a fine-grained and semantic development trend, and information visualization technology makes public opinion monitoring and tracking more intuitive. For example, Xu et al.^7^ constructed the trust function of multi-source data, expressed the multi-source data and decision-making requirements in granularity by using the granularity principle, quantified the degree of association between various types of data by using distance entropy, and promoted the orderly, high-quality and knowledgeable process of emergency decision-making requirements from the perspective of information science with the help of knowledge organization theory and methods, helping to obtain clear and accurate emergency decision-making requirements. In the research of emergency decision-making, Xu et al.^8^ incorporated the public's opinion preference on emergency handling into the decision-making criteria for the emergency decision-making under the social media big data environment, so as to obtain a better decision-making scheme for large groups. Yuan et al.^9^ proposed an emergency risk decision-making method considering regret avoidance for emergencies with multiple possible states by virtue of the thought of regret theory. Fan et al.^10^ proposed an emergency group decision model based on interval-valued fuzzy entropy for the emergency decision problem of multiple network public opinion emergencies with uncertainty indicators. In addition, decision-making hesitation in the context of emergency has a significant impact on the quality of emergency decision-making. Some scholars have begun to explore the moderating role of decision-making hesitation in the path of influencing the quality of decision-making, believing that decision-making experts will have an impact on the final plan regardless of the uncertainty of the choice of the plan or the time delay in making the decision^11^. Xu et al.^12^ focused on the influencing factors and action logic of emergency decision-making quality, and explored the relationship among large group decision-making conflict, risk perception behavior and emergency decision-making quality under different decision-making hesitations. In terms of group decision-making approaches and some applications to emergency management, Li et al.^13^ pointed out that the size of participants and heterogeneous information have important effects on the consensus reaching process in group decision-making, used fuzzy cluster analysis to integrate heterogeneous information for large-scale group decision-making problems. To analyze the influence of the relationship between experts on the decision-making results, Li et al.^14^ proposed a MAGDM with opinion dynamics based on social trust network, considering the opinion dynamics of multi-attribute groups in the decision-making problem.

In view of the incomplete information collection on network public opinion emergencies in a short period of time, decision experts' emergency decisions on network public opinion crisis often involve decision indicators such as sensitivity of public opinion content, public opinion dissemination breadth, and casualties, and it is difficult to make a clear judgment on the situation of emergencies. Since these evaluation attributes are often fuzzy, uncertain and with a certain degree of hesitation, it is reasonable to use the intuitive fuzzy number to evaluate the attributes of the above network public opinion emergencies. The fuzzy set theory proposed by Zadeh in 1965 uses the degree of membership to describe the fuzziness of the objective world, laying the foundation of fuzzy mathematics. Atanassov extended the fuzzy set in 1986, and put forward the theory of intuitionistic fuzzy sets (IFSs)^15^, which can analyze the fuzziness of things more deeply and carefully by introducing concepts such as non-membership degree and hesitation degree, which is the most influential extension and development of fuzzy set theory. As a special intuitionistic fuzzy set on a real number set, intuitionistic fuzzy numbers (IFNs) have the best capability to model ill-known quantities. At present, the research on intuitionistic fuzzy information mainly focuses on the following two aspects: First, in the optimization of quantitative methods for decision analysis, Yuan and Luo^16^ introduced a new decision-making method with IFSs based on novel entropy and evidential reasoning. Rajkumar Verma^17^ presented work develops and studies some parametric information measures under the intuitionistic fuzzy environment, in order to obtain more robust and flexible information measures for IFSs. Zhang et al.^18^ proposed a method to determine the position weight based on discrete normal distribution, constructed a hybrid weighted aggregation operator of IFSs, and integrated the intuitionistic fuzzy evaluation information of each expert about the scheme set. Secondly, in the aspect of optimization and improvement of decision modeling, Joshi and Kumar^19^ proposed to make decisions in intuitionistic fuzzy environment, and established a multi-criteria decision model of intuitionistic fuzzy TOPSIS method based on distance measure and intuitionistic fuzzy entropy (IFE). On this basis, Chen et al.^20^ proposed a new multi-criteria decision-making method in intuitionistic fuzzy environment based on TOPSIS method and similarity, and compared the experimental results of this method with the method in reference^19^ by using a case. The former has zero kill problem and can't get the preference ranking of schemes, which is overcame in the latter and proved to be a more innovative method. Due to the complexity and uncertainty of objective things, it is sometimes difficult to describe the degree of membership and non-membership in the IFSs with precise real values, and it is more appropriate to describe them in the form of interval numbers. For this reason, the IFSs are extended, and the concept of interval-valued intuitionistic fuzzy sets (IVIFSs) is proposed. IFSs and IVIFSs are two important generalizations of fuzzy sets. IFSs best represent these vague phenomena, and admit set operations that do not arise otherwise, because of the functions involved in their definition^21^. This has greatly enriched mathematics and has potential new directions for quantitative studies and applications. During multi-criteria group decision-making (MCGDM), uncertainty arising from linguistic information and varying confidence in estimation is usually available as IVIFSs. Hu et al.^22^ developed an entropy-weighted TOPSIS approach with IVIFSs information, the developed method was then applied to northwest China for supporting the assessment of clean energy-driven desalination-irrigation technology portfolios. With the advantages of taking account of the whole number in the interval and having definite physical meaning, Liu and Jiang^23^ proposed distance measure of IVIFSs shows superiority in measuring uncertainty and imprecision. Rajkumar and Merigó^24^ developed a new flexible method for interval-valued intuitionistic fuzzy decision-making problems with cosine similarity measure. In addition, Nguyen^25^ extended the knowledge measure for the IFSs, and introduced a new interval-valued knowledge measure for the IVIFSs. Liu et al.^26^ proposed a new three-way decision model with IFNs. Muhammad Akram et al.^27^ constructed a new decision-making hybrid model with intuitionistic fuzzy N -soft rough sets.

In recent years, the research on MAGDM based on IFNs has attracted the attention of experts and scholars at home and abroad, and the research results are widely used in many fields such as expert system, logical planning, pattern recognition and machine learning^28,29^. For example, Xu et al.^30^ proposed a large group emergency decision-making method oriented to conflict risk entropy and regret avoidance for uncertain multi-attribute large group emergency risk decision-making. Pang and Song^31^ proposed a MAGDM analysis method based on mixed weight information and risk attitude of decision makers in order to solve the problem of interval intuitionistic uncertain language MAGDM with completely unknown expert weights. In multi-attribute decision-making (MADM), the final decision-making results may present larger differences even when facing the same decision-making problems due to different subjective preferences of decision makers. Zhang et al.^32^ proposed a decision-making method considering expert risk preference for MADM problems with interval-valued intuitionistic fuzzy numbers (IVIFNs) and unknown attribute weights. As the preference of decision-makers is related to both the individual psychological basis of decision-makers and the process of individual judgment and selection, its role and influence on decision-making results should not be underestimated. Zhang et al.^33^ studied the IVIFSs from the viewpoint of the decision makers’ preference. Therefore, it is necessary to study MAGDM from the perspective of decision makers' preference.

Based on the above analysis, under the condition that the existing emergency management departments have limited ability to deal with emergencies, it is a valuable and practical research topic to bring the collective wisdom of decision experts into play and introduce the preference information of decision experts to give an accurate judgment on the crisis severity of network public opinion emergencies. In this paper, according to the intuitionistic fuzzy interval judgment matrix of network public opinion emergencies involving several intuitionistic fuzzy indicators, based on the IFE of emergency decision indicators and expert preference information, an intuitionistic fuzzy emergency group decision model involving emergency department experts is constructed, and the comprehensive crisis value of each network public opinion emergency is calculated, so as to assist the decision-maker in determining the emergency response sequence of the network public opinion emergency event in a timely manner, and the corresponding emergency plan is selected and executed according to the emergency priorities, so as to ensure that the emergency management department is scientific and reasonable in the emergency decision-making and disposal of the network public opinion emergency event.

## Prerequisite knowledge

### Intuitionistic fuzzy number and its algorithm

#### **Definition 1**

If $X$ is a non-empty set, it is called

1 $$ A = \{ < x,\mu_{A} (x),\nu_{A} (x) > |x \in X\} $$

is an intuitionistic fuzzy set, where $\mu_{A} (x)$ and $\nu_{A} (x)$ are the membership and non-membership of the element $x$ in $X$ belonging to $A$, respectively, namely:

$$\mu_{A} :X \to [0,1], \; \; \; x \in X \to \mu_{A} (x) \in [0,1]$$

$$\nu_{A} :X \to [0,1], \; \;\;x \in X \to \nu_{A} (x) \in [0,1]$$

and meeting $$0 \le \mu_{A} (x) + \nu_{A} (x) \le 1$$, $$x \in X$$.

Besides, $\pi_{A} (x) = 1 - \mu_{A} (x) - \nu_{A} (x)$ represents the hesitation or uncertainty of the element $x$ in $X$ belonging to $A$. Obviously, $0 \le \pi_{A} (x) \le 1$^34^.

For convenience, $\alpha = (\mu_{\alpha } ,\nu_{\alpha } )$ is called an intuitionistic fuzzy number.

Where, $$\mu_{\alpha } \in [0,1]$$, $$\nu_{\alpha } \in [0,1]$$, $$\mu_{\alpha } + \nu_{\alpha } \le 1$$.

#### **Definition 2**

If $$\alpha_{1} = (\mu_{{\alpha_{1} }} ,\nu_{{\alpha_{1} }} )$$ and $$\alpha_{2} = (\mu_{{\alpha_{2} }} ,\nu_{{\alpha_{2} }} )$$ are any two IFNs, $$s(\alpha_{i} ) = (\mu_{{a_{i} }} - \nu_{{a_{i} }} )/2 \in [ - 1,1]$$, $$h(\alpha_{i} ) = (\mu_{{a_{i} }} + \nu_{{a_{i} }} )/2 \in [0,1]$$ are called the score value and accuracy of interval values of intuitionistic fuzzy number $$\alpha_{i}$$$$(i = 1,2)$$ respectively^35^, then

1. When $$s(\alpha_{1} ) < s(\alpha_{2} )$$, then $$\alpha_{1} < \alpha_{2}$$;
2. When $s(\alpha_{1} ) = s(\alpha_{2} )$, if $h(\alpha_{1} ) = h(\alpha_{2} )$, then $\alpha_{1} = \alpha_{2}$; if $h(\alpha_{1} ) < h(\alpha_{2} )$, then $\alpha_{1} < \alpha_{2}$; if $h(\alpha_{1} ) > h(\alpha_{2} )$, then $\alpha_{1} > \alpha_{2}$.

When the intuitionistic fuzzy comprehensive crisis values of two network public opinion emergencies are $\alpha_{1}$, $\alpha_{2}$ respectively, and when $\alpha_{1} < \alpha_{2}$, it indicates that the network public opinion emergency $\alpha_{2}$ is more serious than that of $\alpha_{1}$, and the emergency management department is urgently required to give priority to emergency response.

#### **Definition 3**

Let $$WA:R^{n} \to R$$, if

2 $$ WA_{\omega } (a_{1} ,a_{2} , \ldots ,a_{n} ) = \sum\limits_{j = 1}^{n} {\omega_{j} a_{j} } $$

$WA$ is called a weighted average operator, in which $R$ is a set of real numbers, and $\omega = (\omega_{1} ,\omega_{2} , \ldots ,\omega_{n} )^{T}$ is the weight vector of data set $a_{j} (j = 1,2, \ldots ,n)$, $\omega_{j} \in [0,1]$, $\sum\limits_{j = 1}^{n} {\omega_{j} } = 1$^36^.

#### **Definition 4**

Let $\alpha = (\mu_{\alpha } ,\nu_{\alpha } )$, $\alpha_{1} = (\mu_{{\alpha_{1} }} ,\nu_{{\alpha_{1} }} )$ and $\alpha_{2} = (\mu_{{\alpha_{2} }} ,\nu_{{\alpha_{2} }} )$ are any two IFNs^37^, then

1. $$\lambda \alpha = (1 - (1 - \mu_{\alpha } )^{\lambda } ,\nu_{\alpha }^{\lambda } )$$, $$\lambda > 0$$;
2. $$\alpha^{\lambda } = (\mu_{\alpha }^{\lambda } ,1 - (1 - \nu_{\alpha } )^{\lambda } )$$, $$\lambda > 0$$;
3. $$\alpha_{1} \oplus \alpha_{2} = (\mu_{{\alpha_{1} }} + \mu_{{\alpha_{2} }} - \mu_{{\alpha_{1} }} \mu_{{\alpha_{2} }} ,\nu_{{\alpha_{1} }} \nu_{{\alpha_{2} }} )$$;
4. $$\alpha_{1} \otimes \alpha_{2} = (\mu_{{\alpha_{1} }} \mu_{{\alpha_{2} }} ,\nu_{{\alpha_{1} }} + \nu_{{\alpha_{2} }} - \nu_{{\alpha_{1} }} \nu_{{\alpha_{2} }} )$$.

### Distance measure of modified IFSs

Since Atanassov proposed IFSs, there have been abundant research results on the distance and similarity measure of IFSs.

#### **Definition 5**

Let $X$ be a non-empty set, and $\Phi (X)$ be a set of all IFSs on $X$. $d$ is a mapping: $d:(\Phi (X))^{2} \to [0,1]$ , then the distance measure between IFSs $A_{1}$ and $A_{2}$ is $d(A_{1} ,A_{2} )$. Where, $d(A_{1} ,A_{2} )$ meets the conditions^38^:

1. $$0 \le d(A_{1} ,A_{2} ) \le 1$$;
2. $d(A_{1} ,A_{2} ) = 0$, if and only if $A_{1} = A_{2}$;
3. $$d(A_{1} ,A_{2} ) = d(A_{2} ,A_{1} )$$.

#### **Definition 6**

Let $\alpha_{1} = (\mu_{{\alpha_{1} }} ,\nu_{{\alpha_{1} }} )$ and $\alpha_{2} = (\mu_{{\alpha_{2} }} ,\nu_{{\alpha_{2} }} )$ be any two IFNs, and then the distance between IFNs $a_{1}$ and $a_{2}$ is^39^:

3 $$ d(\alpha_{1} ,\alpha_{2} ) = \sqrt {\frac{1}{2}((\mu_{{\alpha_{1} }} - \mu_{{\alpha_{2} }} )^{2} + (\nu_{{\alpha_{1} }} - \nu_{{\alpha_{2} }} )^{2} )} $$

Only two parameters of intuitionistic fuzzy number are considered in the above distance measure: membership degree and non-membership degree. Szmidt and Kacprzyk pointed out that all parameters of intuitionistic fuzzy number should be considered in the distance measure of IFSs, that is, hesitation should not be ignored. Therefore, they modified the distance measure of IFSs.

4 $$ d(\alpha_{1} ,\alpha_{2} ) = \sqrt {\frac{1}{2}((\mu_{{\alpha_{1} }} - \mu_{{\alpha_{2} }} )^{2} + (\nu_{{\alpha_{1} }} - \nu_{{\alpha_{2} }} )^{2} + (\pi_{{\alpha_{1} }} - \pi_{{\alpha_{2} }} )^{2} )} $$

#### **Definition 7**^40^

The mapping $E:IFS(X) \to [0,1]$ is referred to as intuitionistic fuzzy entropy. If it meets the following conditions:

1. $E(A) = 0$, if and only if $A$ is a classic set;
2. $E(A) = 1$, if and only if $\mu_{A} (x_{i} ) = \nu_{A} (x_{i} )$, $\forall x_{i} \in X$;
3. $$E(A) = E(A^{C} )$$;
4. If $A\underline{ \prec } B$, then there is $E\left( A \right) \le E\left( B \right)$.

### The planning of attribute values

The network public opinion emergencies in uncertain network environment contain different emergency decision indicators, which have dimensional differences because of their different meanings. According to the intuitionistic fuzzy interval judgment matrix $R = [r_{ij} ]_{n \times m} = [\mu_{ij} ,1 - \nu_{ij} ]_{n \times m}$, the change range of the target attribute value can be well represented, and the target attribute value is normalized by converting the benefit type and cost type attributes into the interval representation form of the IFSs^41^. Assuming that the measurement value of an attribute given by different dimensions is an interval value $[a_{ij}^{l} ,a_{ij}^{u} ]$, then

Benefit type attribute:

5 $$\mu_{ij} = \frac{{a_{ij}^{l} }}{{\sqrt {\sum\limits_{i = 1}^{n} {(a_{ij}^{u} )^{2} } } }}, \quad \nu_{ij} = 1 - \frac{{a_{ij}^{u} }}{{\sqrt {\sum\limits_{i = 1}^{n} {(a_{ij}^{l} )^{2} } } }} $$

where, $$i = 1,2, \ldots ,n$$; $$j = 1,2, \ldots ,m$$.

Cost type attribute:

6 $$ \mu_{ij} = \frac{{1/a_{ij}^{u} }}{{\sqrt {\sum\limits_{i = 1}^{n} {(1/a_{ij}^{l} )^{2} } } }}, \quad  \nu_{ij} = 1 - \frac{{1/a_{ij}^{l} }}{{\sqrt {\sum\limits_{i = 1}^{n} {(1/a_{ij}^{u} )^{2} } } }} $$

### Establishment of scheme preference model

The following definition of scheme preference is given in consideration of the preference information of experts on schemes and the related characteristics of attributes, attribute importance and the weight of experts themselves involved in the schemes.

#### **Definition 8**

The expert preference for the scheme is the product of the expert weight and the weighted sum of the attribute values and the attribute importance in the scheme, and is recorded as

7 $$ d_{i} = \lambda \sum\limits_{j = 1}^{m} {r_{ij} \omega_{j} } $$

where, $d_{i}$ is preference of the expert for the $i$-th scheme; $\lambda$ is the expert weight, $\lambda \in [0,1]$_,_ determined according to the degree to which experts are recognized in this field. When many experts participate in the scheme decision-making, they will have different preferences for the scheme because of the differences in knowledge structure, professional level and personal experience^42^. If the weight of the $k$-th expert is $\lambda_{k}$,$\lambda_{k} \in [0,1]$, $k = 1,2, \ldots ,p$, in which $p$ is the total number of experts. In order to synthesize the preferences of different experts, the preferences of several experts for different schemes are obtained according to formula (8):

8 $$ d_{i} = \frac{1}{p}\sum\limits_{k = 1}^{p} {\lambda_{k} \sum\limits_{j = 1}^{m} {r_{ij} \omega_{j} } } $$

## Determination of attribute weight and solution of expert weight

In order to comprehensively evaluate the emergency decision-making effect of various alternatives for network public opinion emergencies, the key is to scientifically calculate the attribute weights and expert weights of network public opinion emergencies in addition to obtaining the evaluation values of various attributes of emergency plans.

### Construction of improved intuitionistic fuzzy entropy

In recent years, the traditional IFE theory has been widely concerned, but it can't distinguish IFNs accurately, mainly because it can't fully reflect the influence of hesitation on fuzzy entropy. Yuan and Luo^43^ concluded that the weights rely on not only entropy measures but also the numbers of alternatives and attributes. When the number of attributes increases, the discrepancy between the IFE measures increases. In this paper, the improved IFE is used to weight the attributes of each stage, which lowers the fuzzy degree of the group preference information in the solution.

In reference^44^, the relationship between similarity measure and IFE between IFSs is discussed, and it is proved that IFE and similarity measure can be transformed into each other. The proposed IFE is:

9 $$ E(A) = 1 - \frac{1}{n}\sum\limits_{i = 1}^{n} {|\mu_{A} (x_{i} ) - \nu_{A} (x_{i} )|} $$

In formula (9), the influence of hesitation on intuitionistic fuzzy uncertainty is not considered.

The IFE proposed in reference^45^ is:

10 $$ E(A) = \frac{1}{n}\sum\limits_{i = 1}^{n} {(1 - (\mu_{A} (x_{i} ) + \nu_{A} (x_{i} )))} \cdot \sin (\frac{\pi }{2})(\mu_{A} (x_{i} ) + \nu_{A} (x_{i} )) $$

In formula (10), only the change of $\pi_{A} (x)$ is considered without considering its fuzziness, so IFE cannot be accurately distinguished.

The IFE proposed in reference^46^ is:

11 $$ E(A) = \frac{1}{n}\sum\limits_{i = 1}^{n} {\frac{{\min (\mu_{A} (x_{i} ),\nu_{A} (x_{i} ) + \pi_{A} (x_{i} ))}}{{\max (\mu_{A} (x_{i} ),\nu_{A} (x_{i} ) + \pi_{A} (x_{i} ))}}} $$

By analyzing the formula (11) and combining with the theorem “if and only if $A$ is a Fuzzy set, the entropy value is 0”, it is found that the fuzziness of the fuzzy set itself is ignored in the definition. According to the formula, when $\mu_{A} (x) \ne \nu_{A} (x)$ and $\mu_{A} (x)$ are the same, they are also equal to $E(A)$, but the information reflected by them is not completely equal.

The IFE proposed in reference^47^ is:

12 $$ E(A) = \frac{1}{n}\sum\limits_{i = 1}^{n} {\cot \left(\frac{\pi }{4} + \frac{{|\mu_{A} (x_{i} ) - \nu_{A} (x_{i} )|}}{{4(1 + \pi_{A} (x_{i} ))}}\pi \right)} $$

By analyzing formula (12), it is known that although the influence of hesitation on IFE is considered in this definition, there are still some cases that cannot be well distinguished.

Based on the above analysis, in order to make up for the deficiency of the existing IFE, a class of improved IFE will be constructed in this paper.

For an arbitrary intuitionistic fuzzy set $A = \{ < x_{i} ,\mu_{A} (x_{i} ),\nu_{A} (x_{i} ) > |x_{i} \in X,i = 1,2, \ldots ,n\}$, then:

13 $$ E(A_{j} ) = \frac{1}{n}\sum\limits_{i = 1}^{n} {\cos \frac{{\mu_{A}^{2} (x_{i} ) - \nu_{A}^{2} (x_{i} )}}{2}} \pi $$

Formula (13) can also be written as

14 $$ E(A_{j} ) = \frac{1}{n}\sum\limits_{i = 1}^{n} {\cos \frac{{(\mu_{A} (x_{i} ) - \nu_{A} (x_{i} ))(1 - \pi_{A} (x_{i} ))}}{2}} \pi $$

where, $j = 1,2, \ldots ,m$. According to formula (14), $E(A_{j} )$ contains both the deviation $\mu_{A} (x_{i} ) - \nu_{A} (x_{i} )$ between membership and non-membership, and the information of hesitation $\pi_{A} (x_{i} )$^48^. $(\mu_{A} (x_{i} ) - \nu_{A} (x_{i} ))$ highlights the difference in the degree of uncertainty of IFSs caused by the deviation between membership and non-membership, and $(1 - \pi_{A} (x_{i} ))$ highlights the contribution of hesitation to the unknowns of IFSs^49^. This paper constructs improved IFE, which comprehensively and reasonably describes the fuzziness of IFSs from the two aspects of uncertainty and unknown, and effectively compensates for the existing deficiencies in the definition of IFE.

The attribute weight $\omega_{j}$ is:

15 $$ \omega_{j} = \frac{{1 - E(A_{j} )}}{{m - \sum\nolimits_{j = 1}^{m} {E(A_{j} )} }} $$

The index entropy weight is calculated under the condition that evaluation value of all the alternative emergency decision-making schemes for network public opinion emergencies are given for the each emergency index. It can describe the extent to which each index plays a role in the evaluation of the emergency decision-making scheme. For larger intuitionistic fuzzy information entropy measure of the emergency evaluation index, the corresponding entropy weight is smaller, and the index is less important in the comprehensive evaluation of emergency decision-making.

### Calculation of expert weight

In the evaluation attribute set composed of $m$ attributes and the alternative set composed of $n$ evaluated objects, $\lambda^{k}$ represents the weight w of the $k$-th expert, where, $k = 1,2, \ldots ,p$. The expert's judgment information of each evaluation object is expressed by intuitionistic fuzzy number. In group decision-making problems, the weight of experts depends on the reliability and certainty of expert judgment information, and the fuzziness and uncertainty of judgment information provided by experts can be measured by the IFE of decision matrix. Experts corresponding to systems with large IFE should be given lower weight, otherwise, they should be given higher weight^50^. Based on this idea, the calculation formula of expert weight $\lambda^{k}$ can be obtained.

16 $$ H(A_{j} ) = \frac{1}{n}\sum\limits_{i = 1}^{n} {(1 - (\mu_{A} (x_{i} ) + \nu_{A} (x_{i} )))} \cdot \sin \left(\frac{\pi }{2}\right)\left(\mu_{A} (x_{i} \right) + \nu_{A} (x_{i} )) $$

where, $A_{j}^{k}$ is an intuitionistic fuzzy set composed of the judgment information of $n$ evaluation objects given by the $k$-th expert under the $j$-th attribute. $H(A_{j}^{k} )$ is the IFE of the $k$-th expert under the $j$-th attribute calculated according to the defined IFE formula^51^.

17 $$ H_{k} = \sum\limits_{j = 1}^{m} {\omega_{j} H(A_{j}^{k} )} $$

$H_{k}$ is the weighted IFE, indicating the fuzziness of decision information provided by the $k$-th expert, $0 \le H_{k} \le 1$.

According to the principle of entropy weight method, the expert weight determined by IFE of decision matrix is:

18 $$ \lambda_{k} = \frac{{1 - H_{k} }}{{p - \sum\nolimits_{k = 1}^{p} {H_{k} } }} $$

$\lambda^{k}$ is the weight of the $k$-th expert. Expert weighting formula (18) indicates that it is inversely proportional to $H_{k}$, and the larger $H_{k}$ is, the smaller $\lambda^{k}$ is, and vice versa, which is consistent with the idea of expert weighting put forward in this paper, and reflects the inverse relationship between expert weighting and the fuzziness of judgment information given by it, and ensures $0 \le \lambda_{k} \le 1$ and $\sum\limits_{k = 1}^{p} {\lambda_{k} } = 1$ . Different from the subjective method of determining experts' weights according to their importance, formula (18) is a more reasonable and effective objective expert weighting method, which uses the fuzziness and uncertainty of information provided by experts instead of the traditional deviation between individual decision-making information and group decision-making information^52^.

## MAGDM method based on intuitionistic fuzzy entropy and preference information of decision makers

### Problem description

For a certain MAGDM problem, let $A = \{ A_{1} ,A_{2} , \ldots ,A_{n} \}$ be a scheme set, and then the decision makers compare $n$ schemes pairwise and construct the intuitionistic fuzzy decision-making matrix $R = [r_{ij} ]_{n \times m}$, in which $r_{ij} = [\mu_{ij} ,1 - \nu_{ij} ]$ , $i = 1,2, \ldots ,n$; $j = 1,2, \ldots ,m$. $\mu_{ij}$ represents the preference of decision makers for $x_{i}$ when comparing schemes $x_{i}$ and $x_{j}$, $\nu_{ij}$ represents the preference of decision makers for $x_{j}$ , and $1 - \mu_{ij} - \nu_{ij}$ represents the hesitation of decision makers. If $\mu_{ij} \in [0,1]$, $\nu_{ij} \in [0,1]$, $0 \le \mu_{ij} + \nu_{ij} \le 1$. In this paper, scheme set $A$ is a set of alternative schemes composed of $n$ simultaneous network public opinion emergencies, of which $A_{i}$ is the $i$-th scheme, $i = 1,2, \ldots ,n$; the evaluation attribute set is $C = \{ C_{1} ,C_{2} , \ldots ,C_{m} \}$, which is composed of $m$ attributes, in which $C_{j}$ is the $j$-th attribute of the scheme, $j = 1,2, \ldots ,m$; $Exp = \{ Exp_{1} ,Exp_{2} , \ldots ,Exp_{p} \}$ is the evaluation group composed of $p$ decision makers. The decision makers respectively collect objective data and judge subjectively the crisis degree of network public opinion emergencies, $\lambda^{k}$ is the weight of the $k$-th decision maker, meeting $0 \le \lambda_{k} \le 1$ and $\sum\limits_{k = 1}^{p} {\lambda_{k} } = 1$. $\omega_{j}$ is the weight of the decision makers on the evaluation attribute $C_{j}$,$j = 1,2, \ldots ,m$ , meeting $\omega_{j} \ge 0$ and $\sum\limits_{j = 1}^{m} {\omega_{j} } = 1$. $r_{ij}$ is the evaluation value of the decision makers on the alternative $A_{i}$ under the attribute $C_{j}$, and the corresponding decision matrix is $R = [r_{ij} ]_{n \times m}$. Considering that different dimensions among attributes in the decision matrix $R$ will affect the decision results, it is necessary to normalize the evaluation information in the decision matrix so as to obtain a normalized intuitionistic fuzzy decision matrix $A = [r_{ij} ]_{n \times m}$.

### Optimization model of attribute weight

#### **Definition 9**

Let the standardized intuitionistic fuzzy interval judgment matrix be $A = [r_{ij} ]_{n \times m}$, in which $r_{ij} = [\mu_{ij} ,1 - \nu_{ij} ]$. If the norm of any two interval numbers in a matrix is $||r_{ij} - r_{kj} || = |\mu_{ij} - \mu_{kj} | + |(1 - \nu_{ij} ) - (1 - \nu_{kj} )|$, $d(r_{ij} ,r_{kj} ) = ||r_{ij} - r_{kj} ||$ is called the proximity between elements $r_{ij}$ and $r_{kj}$ in the intuitionistic fuzzy interval judgment matrix^53^.

#### **Definition 10**

Let the standardized intuitionistic fuzzy interval judgment matrix be $A = [r_{ij} ]_{n \times m}$, the positive ideal solution of each attribute is $r_{j}^+ = [\mu_{j}^{ + }, 1-\nu_{j}^{ + } ]$ and

$$ \mu_{j}^{ + } = \max \{ \mu_{ij}^{ + } ,i = 1,2, \ldots ,n;j = 1,2, \ldots ,m\} $$

$$ 1 - \nu_{j}^{ + } = \min \{ 1 - \nu_{ij}^{ + } ,i = 1,2, \ldots ,n;j = 1,2, \ldots ,m\} $$

For attribute $C_{j}$, the deviation between the evaluation value and the ideal solution of each scheme is:

19 $$ D_{i} (\omega ) = \sum\limits_{j = 1}^{m} {d^{2} (r_{i}^{ + } ,r_{ij} )} \omega_{j}^{2} $$

The total deviation between the evaluation values of all schemes and the ideal solution should be minimized in the selection of attribute weights, i.e., the following optimal attribute weight constraint optimization model should be met:

20 $$ \left\{ {\begin{array}{*{20}l} {\min H_{1} (\omega ) = \sum\limits_{i = 1}^{n} {D_{i} (\omega )} = \sum\limits_{i = 1}^{n} {\sum\limits_{j = 1}^{m} {d^{2} (r_{j}^{ + } ,r_{ij} )\omega_{j}^{2} } } } \hfill \\ {\sum\limits_{j = 1}^{m} {\omega_{j} = 1} ,\omega_{j} \ge 0,j = 1,2, \ldots ,m} \hfill \\ \end{array} } \right. $$

In addition, it is hoped that the subjective preference of decision makers will play a guiding role in decision-making and ranking. Due to a certain gap between the subjective preference and the objective preference of the decision-maker, in order to make the decision reasonable, the total deviation between objective preference and subjective preference should be as small as possible in the selection of attribute weight $\omega$. To build an optimization model for obtaining attribute weights considering the preference of decision makers, the following attribute constraint optimization model should be met:

21 $$ \left\{ {\begin{array}{*{20}l} {\min H_{2} (\omega ) = \sum\limits_{i = 1}^{n} {\sum\limits_{j = 1}^{m} {d^{2} (r_{ij} ,d_{i} )\omega_{j}^{2} } } } \hfill \\ {\sum\limits_{j = 1}^{m} {\omega_{j} = 1} ,\omega_{j} \ge 0,j = 1,2, \ldots ,m} \hfill \\ \end{array} } \right. $$

The linear weighting method is used to synthesize the programming model, and the programming models described by formula (20) and formula (21) are synthesized into the optimization model with the following attribute weights:

22 $$ \left\{ {\begin{array}{*{20}l} {\min H(\omega ) = \alpha \sum\limits_{i = 1}^{n} {\sum\limits_{j = 1}^{m} {d^{2} (r_{j}^{ + } ,r_{ij} )\omega_{j}^{2} } } + \beta \sum\limits_{i = 1}^{n} {\sum\limits_{j = 1}^{m} {d^{2} (r_{ij} ,d_{i} )\omega_{j}^{2} } } } \hfill \\ {\sum\limits_{j = 1}^{m} {\omega_{j} = 1} ,\omega_{j} \ge 0,j = 1,2, \ldots ,m;\alpha + \beta = 1} \hfill \\ \end{array} } \right. $$

In formula (22), $\alpha$ and $\beta$ are the weight coefficients for adjusting two kinds of programming models. When $\alpha = 1$ and $\beta = 0$, that is, the programming model $(M - 1)$ described by formula (20), the method of determining attribute weights at this time is based on the ideal attribute value method. When $\alpha = 0$ and $\beta = 1$, that is, the programming model $(M - 2)$ described by formula (21), the method to determine the attribute weight at this time is the preference assignment method in which the decision maker has preference information for the scheme. If $\alpha ,\beta \in [0,1]$, the method for determining the weight of the target attributes by the integrated programming model $(M - 3)$ described in formula (22) can reflect both subjective and objective information.

### Steps for decision making

In this paper, a MAGDM method based on intuitionistic fuzzy preference information is proposed under the condition that the decision-maker weights and attribute weights are completely unknown, and the following specific algorithm steps are given.

Step 1: In view of the simultaneous outbreak of network public opinion emergencies in a certain area $\{ A_{1} ,A_{2} , \ldots ,A_{n} \}$, the emergency management department invited several experts to evaluate $n$ emergencies from $m$ aspects. The intuitionistic fuzzy interval judgment matrix $R = [r_{ij} ]_{n \times m}$ is established based on the quantitative analysis of the attribute values of evaluation schemes. Because there may be different measurement standards among attributes, and attributes can be divided into benefit type and cost type, it is necessary to eliminate the differences in dimensions, units and types of attributes before making decisions. Therefore, formulas (5) and (6) are used to normalize them and construct a normalized intuitionistic fuzzy interval judgment matrix $A = [r_{ij} ]_{n \times m} = [\mu_{ij} ,1 - \nu_{ij} ]_{n \times m}$.
Step 2: The improved IFE construction method proposed in “Construction of improved intuitionistic fuzzy entropy” section is used to calculate the attribute weights $\omega_{j}$ in the intuitionistic fuzzy emergency decision matrix.
Step 3: The weight $\lambda_{k}$ ($k = 1,2, \ldots ,p$) of experts are obtained based on the decision matrix intuitionistic fuzzy entropy using the calculation method of expert weights in “Calculation of expert weight” section.
Step 4: After obtaining the standardized intuitionistic fuzzy emergency decision matrix and the weight of each evaluation index, According to formula (8), the overall preference $d_{i}$ of many experts for different schemes is calculated.
Step 5: According to the standardized intuitionistic fuzzy emergency decision matrix $A = [r_{ij} ]_{n \times m}$, the positive and negative ideal solutions of each target attribute are determined.
  The positive ideal solution $r_{j}^{ + }$ is:
  $$ r_{j}^{ + } = \left\{ {\begin{array}{*{20}c} {\mathop {\max }\limits_{i} r_{ij} = (\mathop {\max }\limits_{i} \mu_{ij}^{ + } ,\mathop {\min }\limits_{i} \nu_{ij}^{ + } ),} & {j \in \Omega_{1} } \\ {\mathop {\min }\limits_{i} r_{ij} = (\mathop {\min }\limits_{i} \mu_{ij}^{ + } ,\mathop {\max }\limits_{i} \nu_{ij}^{ + } ),} & {j \in \Omega_{2} } \\ \end{array} } \right. $$
  The negative ideal solution $r_{j}^{ - }$ is:
  $$ r_{j}^{ - } = \left\{ {\begin{array}{*{20}c} {\mathop {\min }\limits_{i} r_{ij} = (\mathop {\min }\limits_{i} \mu_{ij}^{ - } ,\mathop {\max }\limits_{i} \nu_{ij}^{ - } ),} & {j \in \Omega_{1} } \\ {\mathop {\max }\limits_{i} r_{ij} = (\mathop {\max }\limits_{i} \mu_{ij}^{ - } ,\mathop {\min }\limits_{i} \nu_{ij}^{ - } ),} & {j \in \Omega_{2} } \\ \end{array} } \right. $$
  where, $i = 1,2, \ldots ,n$; $j = 1,2, \ldots ,m$. $\Omega_{1}$ and $\Omega_{2}$ are subscript sets of benefit-type and cost-type attributes.
Step 6: The formula (22) is used to solve the integrated programming model and get the optimal weight distribution of attributes $\omega^{\prime}_{j}$.
Step 7: The synthesized attribute value $z_{i}$ of the crisis severity of all network public opinion emergencies is calculated by formula (23).
  23 $$ z_{i} = \sum\limits_{j = 1}^{m} {r_{ij} \omega^{\prime}_{j} } $$

Finally, according to the value of $z_{i}$, multiple emergencies that occur simultaneously are ranked. The larger the value is, the more serious the crisis of the corresponding network public opinion emergencies will be and the greater the social impact will be. In the case of limited personnel and resources, decision makers should give priority to dealing with emergencies with the most serious crisis. The evolution of emergencies displays dynamic, complex, systematic characteristics. Emergency management departments need take effective response measures within a short period of time to prevent the spread and escalation of the situation, thereby reducing the hazards and subsequent impacts in a timely manner.

## Instance analysis

Real-time collection of online public opinion data from domestic mainstream portals such as WeChat, Weibo, Baidu Index and Zhihu was conducted in a certain area through the public opinion monitoring department. Analysis by relevant technical personnel revealed that five simultaneous online public opinion emergencies $\{ A_{1} ,A_{2} ,A_{3} ,A_{4} ,A_{5} \}$ might occur. Due to the limited resources, facilities and emergency personnel in this area, public opinions should be handled in the order from the most serious to the least. Several emergency experts were invited to conduct interviews, investigations and analysis, and 8 key decision-making evaluation indicators were identified: proneness of emergencies $(C_{1} )$, speed of emergency spread $(C_{2} )$, attention to public opinion $(C_{3} )$, breadth of public opinion dissemination $(C_{4} )$, sensitivity of public opinion content $(C_{5} )$, tendentiousness of public opinion attitude $(C_{6} )$, economic losses $(C_{7} )$, and casualties $(C_{8} )$^10^. Subsequently, the staff of the network public opinion crisis monitoring department in the region and emergency experts from different industries were organized to conduct initial evaluation on the above 8 key decision-making evaluation indicators. The obtained raw data are shown in Table 1.

**Table 1:** Original data of decision-making evaluation indicators of network public opinion emergencies.

	*C*_1_	*C*_2_	*C*_3_	*C*_4_	*C*_5_	*C*_6_	*C*_7_	*C*_8_
*A*_1_	[0.55,0.72]	[13, 25]	[0.3,0.6]	[300,320]	[700,800]	[100,150]	[6,10]	[0.5,0.6]
*A*_2_	[0.52,0.63]	[40,58]	[0.7,0.8]	[290,320]	[800,850]	[310,350]	[12,16]	[0.7,0.9]
*A*_3_	[0.26,0.34]	[59,82]	[0.6,0.7]	[100,150]	[1500,1700]	[350,500]	[2,4]	[0.5,0.8]
*A*_4_	[0.34,0.50]	[36,58]	[0.6,0.8]	[510,550]	[1000,1300]	[280,350]	[12,18]	[0.7,0.8]
*A*_5_	[0.13,0.17]	[32, 49]	[0.4,0.6]	[810,860]	[3000,3500]	[250,300]	[18,25]	[0.2,0.6]

1. Since all the emergency decision-making indicators of network public opinion emergencies are benefit-type attributes, whose evaluation values were substituted into formulas (5)–(6) and normalized to get the normalized intuitionistic fuzzy emergency decision-making matrix $A = [r_{ij} ]_{n \times m}$, as shown in Table 2.

**Table 2:** Standardized intuitionistic fuzzy emergency decision matrix.

	*C*_1_	*C*_2_	*C*_3_	*C*_4_	*C*_5_	*C*_6_	*C*_7_	*C*_8_
*A*_1_	[0.481,0.819]	[0.101,0.287]	[0.190,0.497]	[0.266,0.305]	[0.164,0.219]	[0.128,0.247]	[0.165,0.392]	[0.298,0.487]
*A*_2_	[0.454,0.717]	[0.312,0.667]	[0.444,0.662]	[0.257,0.305]	[0.188,0.232]	[0.398,0.576]	[0.330,0.627]	[0.418,0.730]
*A*_3_	[0.227,0.387]	[0.460,0.942]	[0.380,0.579]	[0.089,0.143]	[0.352,0.465]	[0.449,0.823]	[0.055,0.157]	[0.298,0.649]
*A*_4_	[0.297,0.569]	[0.280,0.667]	[0.380,0.662]	[0.453,0.524]	[0.234,0.355]	[0.359,0.576]	[0.330,0.705]	[0.418,0.649]
*A*_5_	[0.114,0.193]	[0.249,0.563]	[0.253,0.497]	[0.719,0.820]	[0.703,0.957]	[0.321,0.494]	[0.495,0.979]	[0.119,0.487]
2. The attribute weights in emergency decision matrix were calculated by improved IFE construction method. Firstly, the IFE $E(A_{j} )$ of each emergency decision evaluation index was calculated according to the emergency decision matrix by using formula (14).
  $$\begin{aligned} &E(A_{1} ) = 0.8665, \; E(A_{2} ) = 0.9270, \; E(A_{3} ) = 0.9775, \; E(A_{4} ) = 0.7462, \; E(A_{5} ) = 0.7559, \\ & E(A_{6} ) = 0.9160, \; E(A_{7} ) = 0.8452, \; E(A_{8} ) = 0.9739\end{aligned}$$
  Then, the weight vector $\omega_{j}$ of each emergency decision evaluation indicator was calculated by the information entropy measure formula as follows:
  $$ \omega_{j} = (0.135,0.074,0.023,0.256,0.246,0.085,0.156,0.026) $$
3. According to formulas (16)–(18), the weighted IFE of experts was calculated, and the weights of experts were determined by the IFE of decision matrix.
  The IFE $H(A_{j} )$ of experts under each attribute is:
  $$ \begin{aligned} & H(A_{1} ) = 0.2026,\;  H(A_{2} ) = 0.2847, \; H(A_{3} ) = 0.2294, \; H(A_{4} ) = 0.0622, \; H(A_{5} ) = 0.1126 \\ & H(A_{6} ) = 0.1938, \; H(A_{7} ) = 0.2480, \; H(A_{8} ) = 0.2557 \end{aligned}$$
  A total of 4 experts participated in the decision making. Due to the space limitation, the operation process of expert joint decision-making will not be described in more detail. The weighted IFE of several experts $H_{k}$ is:
  $$ H_{k} = (0.159,0.150,0.161,0.174) $$
  The expert weight $\lambda_{k}$ is:
  $$ \lambda_{k} = (0.251,0.253,0.250,0.246) $$
4. The overall preference $d_{i}$ of a plurality of experts on each network public opinion emergency was calculated according to the formula (8).
  $$ \begin{gathered} d_{i} = \{ [0.212,0.362],[0.103,0.230],[0.037,0.066],[0.457,0.537],[0.404,0.548], \hfill \\ [0.140,0.230],[0.215,0.446],[0.041,0.079]\} \hfill \\ \end{gathered} $$
5. According to the normalized intuitionistic fuzzy emergency decision matrix $A = [r_{ij} ]_{n \times m}$, the positive and negative ideal solutions of each target attribute were determined.
  The positive ideal solution $r_{j}^{ + }$ is:
  $$ \begin{gathered} r_{j}^{ + } = \{ [0.481,0.193],[0.460,0.287],[0.444,0.497],[0.719,0.143],[0.703,0.219], \hfill \\ [0.449,0.247],[0.495,0.157],[0.418,0.487]\} \hfill \\ \end{gathered} $$
  The negative ideal solution $r_{j}^{ - }$ is:
  $$ \begin{gathered} r_{j}^{ - } = \{ [0.114,0.819],[0.101,0.942],[0.190,0.662],[0.089,0.820],[0.164,0.957], \hfill \\ [0.128,0.823],[0.055,0.979],[0.119,0.730]\} \hfill \\ \end{gathered} $$
6. The comprehensive programming model was solved to get the optimal weight allocation $\omega^{\prime}_{j}$ of the target attributes under different model parameters, and then the synthesized attribute value $z_{i}$ of the crisis severity of the above five network public opinion emergencies was calculated according to step 7. The comparison results are shown in Table 3.

**Table 3:** Optimal weights of attributes under different model parameters and synthesized attribute values of target crisis severity.

Model parameter (*α*, *β*)	Attribute optimal weight (*w*_*j*_)	Comprehensive attribute value (*z*_*i*_)
M-1 (*α* = 1, *β* = 0)	(0.05,0.10,0.40,0.03,0.04,0.10,0.03,0.25)	(0.875,0.972,0.948,0.991,0.914)
M-3 (*α* = 0.8, *β* = 0.2)	(0.10,0.10,0.24,0.10,0.10,0.11,0.05,0.20)	(0.840,0.937,0.888,0.982,0.863)
M-3 (*α* = 0.5, *β* = 0.5)	(0.12,0.10,0.20,0.10,0.10,0.14,0.10,0.14)	(0.826,0.938,0.857,0.982,0.856)
M-3 (*α* = 0.2, *β* = 0.8)	(0.20,0.10,0.10,0.13,0.13,0.14,0.12,0.08)	(0.809,0.921,0.817,0.976,0.810)
M-2 (α = 0, *β* = 1)	(0.20,0.10,0.04,0.20,0.20,0.10,0.12,0.04)	(0.787,0.883,0.776,0.966,0.777)

According to Table 3, when the parameters of the integrated model are selected differently, the obtained optimal weight allocation of attributes is different. Among them, the weights of attributes $C_{3}$ (public opinion attention) and $C_{8}$ (casualties) are gradually reduced from model $(M - 1)$ to $(M - 2)$, which is due to the increasing role of experts' subjective preference information in the model, but the weight of this attribute is always the largest compared with other attributes, indicating that this attribute plays the greatest role in decision-making results, and the weight distribution of other attributes has changed partially due to model transformation.

As to the comprehensive ranking of crisis severity of network public opinion emergencies, when the model is biased towards objective ranking mode $(M - 1)$, the crisis severity of scheme (emergency) $A_{2}$ and $A_{4}$ is equally the most serious; when the model is biased towards subjective ranking model $(M - 2)$, only scheme (emergency) $A_{4}$ becomes obviously the most serious due to the role of experts' subjective preference for the crisis severity of schemes in the model. However, scheme $A_{1}$ always has the smallest degree of crisis under any circumstances, and other schemes have different ranking results due to the different emphasis of the model.

When the proportion of objective information and subjective preference is the same, that is, $\alpha = 0.5$, $\beta = 0.5$ , after evaluating the crisis severity of the network public opinion emergencies, the ranking result is $A_{4} \succ A_{2} \succ A_{3} \succ A_{5} \succ A_{1}$, i.e., network public opinion emergency $A_{4}$ have the greatest degree of crisis, followed by emergency $A_{2}$, while emergency $A_{1}$ has the smallest degree of crisis. In the attribute weights of emergency decision matrix calculated by improved intuitionistic fuzzy entropy, the weights of public opinion dissemination breadth $(C_{4} )$, sensitivity of public opinion content $(C_{5} )$ and economic losses $(C_{7} )$ are 0.256, 0.246 and 0.156, respectively, ranking the top three among the eight attributes. Although the evaluation values of interval-valued attributes $C_{4} ,C_{5} ,C_{7}$ do not reach the maximum value in the network public opinion emergency $A_{4}$, the attribute weight value comes first. Combined with the corresponding values and weights of other attributes, the comprehensive hazard interval value of the emergency $A_{4}$ is calculated to be the largest by the weighted aggregation method. Judging from this, the network public opinion emergency will have a huge impact on society. If not handled properly, it will lead to a serious public opinion crisis, which is likely to induce bad emotions among the public and trigger violations and excesses by the masses, thus posing a threat to social stability. Therefore, in the case of limited human and material resources, decision makers should give priority to $A_{4}$ public opinion emergency, and formulate corresponding emergency plans, and then deal with other events with low severity in turn, the emergency rescue plan can be revised and improved with the development of the situation.

The research results have been recognized by experts in the field of emergency decision-making, which provides a scientific way for emergency management departments to respond quickly and dynamically deal with online public opinion. At the same time, it also provides an effective scientific method for solving the problem of multi-attribute intuitionistic fuzzy group decision-making. Thus it is clear that the method proposed in this study the related research results has the following advantages over:

1. Compared with the method in reference^54^, based on the fuzziness of information in the emergency decision environment, the improved IFE construction method is used to comprehensively and effectively depict the fuzzy information from two aspects of uncertainty and unknown, which improves the accuracy and objectivity of the decision results to a certain extent. In the former, fuzzy linguistic variables are aggregated by fuzzy ordered weighted average operator, and language is transformed into fuzzy numbers by sampling survey. Therefore, the accuracy and objectivity of decision-making results are improved in the latter as compared with the former.
2. Compared with the former^54^, according to the characteristics of experts' preference for schemes, a linear programming model of scheme preference among experts' weights, attribute values and attribute importance in schemes is constructed, and the comprehensive preference of several experts for different schemes is given. In the former, deblurring is used to construct the expert evaluation matrix, and three behavioral decision models are used to determine the acceptance domain and the rejecting domain without taking into account such factors as the preference information of experts on the scheme. Therefore, the decision result obtained by the latter is more objective and convincing than the former.
3. This study presents an emergency crisis assessment method for MAGDM based on IFSs, and discusses the application of MAGDM with intuitionistic fuzzy preference information in the crisis assessment of network public opinion emergencies. However, some of the existing research results, such as literature^8^ and literature^55^, mainly conduct public opinion analysis and post-disaster analysis on social media data after emergencies, which are not really integrated with emergency decision-making.
4. In the latter, an attribute optimization weight model is constructed, which not only considers the objective information of each attribute value in the evaluation scheme (emergency), but also takes into account the subjective preference of experts to judge the crisis degree of the scheme based on their professional level and rich experience, which reflects the influence of subjective and objective information on the crisis severity evaluation of network public opinion emergencies, and provides a more powerful mathematical model for emergency decision-making in complex environment. However, the former^56^ only determines the attribute weight based on the improved IFE formula, and does not consider determining the expert weight according to the importance of the experts.

## Conclusions

In this era of information explosion, information dissemination and opinion interaction are faster than ever before, and the expression demands of network public opinion are more and more diverse. If not correctly guided, negative network public opinion will pose a greater threat to social and public safety. Therefore, it is of great practical significance to strengthen the timely monitoring and effective guidance of network public opinion and to actively resolve the crisis of network public opinion to maintain social stability and promote national development, as well as to create a harmonious society. Due to the urgency and complexity of the emergency decision making of network public opinion emergencies, the obtained decision information and the judgment on the information are fuzzy and uncertain, so the MAGDM problem with the evaluation value as the intuitionistic fuzzy number is discussed in this paper. In order to reflect the objectivity of decision-making and give consideration to the subjective preference of decision-makers, the improved IFE construction method is used to determine attribute weights and expert weights, which can minimize the loss of decision-making information and reflect the authenticity of experts' wishes. In addition, the optimization model of attribute weights is constructed considering both the objective information of the evaluation of the emergency crisis degree by attribute values and the subjective preference of experts for judging the emergency crisis degree based on their professional level and rich experience. This research presents an emergency crisis assessment method for MAGDM based on IFSs, and discusses the application of MAGDM with intuitionistic fuzzy preference information in the crisis assessment of network public opinion emergencies. Through target crisis assessment examples, it is verified that the method can effectively avoid the deviation caused by the influence of external environmental factors and the subjective experience of decision-makers. With regard to future research planning, in the current complex and dynamic network public opinion environment, building a dynamic evolution model of network public opinion based on multi-perspectives combined with multi-case big data can provide a more forward-looking understanding of the evolution law of network public opinion emergencies, and provide a methodological basis for public opinion participants and regulatory authorities to grasp the evolution trend of public opinion in time. In the actual application process, relevant databases should be established, more experience should be accumulated. It is possible to select appropriate model parameters to improve the performance of the dynamic evolution model in intuitionistic fuzzy emergency group decision-making. The characteristics of typical emergencies can be reflected through knowledge representation norms. The effectiveness of knowledge reasoning rules related to emergency decision-making, and how to quickly and accurately match the knowledge base with emergency problems in the selection of emergency decision-making methods can serve as a future research targets.
